# Thyroid-Stimulating Hormone Inhibits Adipose Triglyceride Lipase in 3T3-L1 Adipocytes through the PKA Pathway

**DOI:** 10.1371/journal.pone.0116439

**Published:** 2015-01-15

**Authors:** Dongqing Jiang, Shizhan Ma, Fei Jing, Chao Xu, Fang Yan, Aihong Wang, Jiajun Zhao

**Affiliations:** 1 Department of Endocrinology and Metabolism, Shandong Provincial Hospital affiliated to Shandong University, Jinan, Shandong, China; 2 Institute of Endocrinology, Shandong Academy of Clinical Medicine, Jinan, Shandong, China; 3 Department of Endocrinology and Metabolism, The Second Hospital of Shandong University, Jinan, Shandong, China; 4 Department of Cardiology, Shandong Provincial Hospital affiliated to Shandong University, Jinan, Shandong, China; University of Warwick – Medical School, UNITED KINGDOM

## Abstract

Thyroid-stimulating hormone (TSH) has been shown to play an important role in the regulation of triglyceride (TG) metabolism in adipose tissue. Adipose triglyceride lipase (ATGL) is a rate-limiting enzyme controlling the hydrolysis of TG. Thus far, it is unclear whether TSH has a direct effect on the expression of ATGL. Because TSH function is mediated through the TSH receptor (TSHR), TSHR knockout mice (*Tshr*-/- mice) (supplemented with thyroxine) were used in this study to determine the effects of TSHR deletion on ATGL expression. These effects were verified in 3T3-L1 adipocytes and potential underlying mechanisms were explored. In the *Tshr*-/- mice, ATGL expression in epididymal adipose tissue was significantly increased compared with that in *Tshr*+/+ mice. ATGL expression was observed to increase with the differentiation process of 3T3-L1 preadipocytes. In mature 3T3-L1 adipocytes, TSH significantly suppressed ATGL expression at both the protein and mRNA levels in a dose-dependent manner. Forskolin, which is an activator of adenylate cyclase, suppressed the expression of ATGL in 3T3-L1 adipocytes. The inhibitory effects of TSH on ATGL expression were abolished by H89, which is a protein kinase A (PKA) inhibitor. These results indicate that TSH has an inhibitory effect on ATGL expression in mature adipocytes. The associated mechanism is related to PKA activation.

## Introduction

TSH is a tropic hormone released by the pituitary gland that primarily acts on the thyroid gland via binding to its receptor and plays essential roles in thyroid development and function. Accumulating evidence has shown that, in addition to the thyroid gland, TSH also acts in many other tissues including fat tissue. Clinical studies have indicated a positive association between thyroid-stimulating hormone (TSH) and serum triglyceride (TG) levels [1, 2]. TSH receptor (TSHR) knockdown has been shown to block the differentiation of 3T3-L1 preadipocytes [3]. It is now accepted that TSH can accelerate the differentiation of 3T3-L1 cells. In mature adipocytes, TSH has been reported to regulate the lipolysis of TGs in an acute manner [4]. However, it is not known whether TSH has more long-term effects on lipolysis, and the precise underlying mechanisms are not yet fully understood.

Adipose triglyceride lipase (ATGL) is a recently identified rate–limiting enzyme that primarily catalyzes the hydrolysis of TG to generate diacylglycerol and free fatty acid (FFAs) [5–8]. In addition, ATGL also exhibits weak diglyceride (DG) lipase, phospholipase and transacylase activities. This enzyme is predominantly expressed in adipose tissue. It is also expressed at low levels in non-adipose tissues including cardiac muscle, skeletal muscle and liver. ATGL-null mice display the accumulation of neutral lipids in most tissues due to impaired lipolysis. Excessive lipid accumulation in the heart has been shown to cause cardiac dysfunction and premature death [9]. Adipose triacylglycerol lipase deletion has been demonstrated to alters whole-body energy metabolism and impair exercise performance in mice [10].

Structurally, ATGL contains multiple phosphorylation sites, and Ser-406 has been identified to be a target residue for protein kinase A (PKA). The PKA-mediated phosphorylation of ATGL Ser-406 has been shown to moderately increase ATGL-mediated lipolysis [11, 12].

Taken together, the above studies indicate that both TSH and ATGL are involved in the regulation of TG metabolism and lipolysis. However, a direct link between TSH and ATGL has not been documented. The aim of the present study was to investigate the effect of TSH on ATGL expression both *in vitro* and in *vivo*. The potential mechanism underlying these effects was also explored.

## Materials and Methods

### Animals and experimental procedures

*Tshr-/-* mice (stain name: B6; 129S1-*Tshr* ^tm1Rmar^/J) were purchased from Jackson Laboratory (Bar Harbor, ME). *Tshr-/-* mice and *Tshr+/+* mice were obtained by breeding heterozygote (*Tshr+/-*) mice. All animals were housed in a temperature-controlled room (22–23°C) under diurnal lightning conditions with free access to food and water. The *Tshr-/-* mice were fed a diet containing 100 ppm desiccated thyroid extract (Sigma-Aldrich, St. Louis, MO) from 21 days of age [13]. After 3 weeks, the *Tshr-/-* mice and age-matched wild-type mice were fasted for 12 h, and blood samples were collected for measurements of serum total T4 (TT4) levels. For additional experiments, 8-week-old wild-type and *Tshr-/-* mice were starved for 8 h, and weighed. Then, the mice were sacrificed by decapitation under anesthesia with pentobarbital sodium. Epididymal adipose tissues were collected, weighed and frozen in liquid nitrogen until use. All animal experiments were in compliance with the relevant federal guidelines and institutional policies, and the animal protocol was approved by the Animal Care and Use Committee of Shandong Provincial Hospital affiliated with Shandong University (approval number: No. 2014–073). All surgical procedures were performed under sodium pentobarbital anesthesia, and all efforts were made to minimize suffering.

### Cell culture and initiation of differentiation

3T3-L1 cells were obtained from the American Type Culture Collection (ATCC). The 3T3-L1 cells were cultured in Dulbecco’s modified Eagle’s medium (DMEM, Gibco BRL, Gaithersburg, MD, USA) supplemented with 10% (v/v) newborn calf serum(NBS, Gibco), 100 U/mL penicillin, and 0.1 mg/mL streptomycin (KeyGEN, Nanjing, China) in a humidified 5% CO_2_ incubator at 37°C. To induce differentiation, confluent preadipocytes(this day was marked as D0, the second day was marked as D1, and so on) were treated for 2 days with 0.5 mmol/L isobutylmethylxanthine (IBMX, Sigma-Aldrich), 2.5 μmol/L dexamethasone (Dex, Sigma-Aldrich) and 8.7 μmol/L insulin (Sigma-Aldrich) in DMEM containing 10% fetal calf serum (FCS, Gibco), followed by treatment for another 2 days with insulin (10 μM) alone in DMEM containing 10% FCS. Subsequently, the cells were replenished with DMEM containing 10% FCS every other day. On day 12, approximately 90% of the cells had differentiated into adipocytes.

### Cell stimulation

Differentiated 3T3-L1 adipocytes were starved in serum-free DMEM for 1 h before stimulation. The cells were then treated with recombinant bovine TSH (bTSH, St Louis, MO, USA), forskolin (Sigma-Aldrich) and H89 (Sigma-Aldrich) according to the experimental design.

### Western blot analysis

Adipose tissue samples were homogenized in RIPA lysis buffer containing protease inhibitors. Protein concentrations were determined by the BCA method. Proteins (110 μg) were separated on 10% SDS-PAGE gels and transferred to a PVDF membrane (Millipore, USA).The membranes were blocked in 5% (w/v) non-fat milk for 1 h and then incubated with rabbit anti-ATGL (Cell Signaling Beverly, MA, USA,1:1000 dilution), rabbit anti-GAPDH (CW Biotech, Beijing, China, 1:3000 dilution) or mouse anti-β-actin (Proeintech, Chicago, IL, USA, 1:2000 dilution) primary antibodies overnight at 4°C. Subsequently, the membranes were incubated with peroxidase-conjugated anti-rabbit or anti-mouse secondary antibody for 1 h at room temperature. After washing with TBST, the immune complexes were detected with the Alpha Q Chemiluminescence System and exposed to film. The relative intensity of the target protein to GAPDH or to β-actin in the same sample was analyzed with Alpha Q software.

### RNA extraction and quantitative real-time PCR analysis

Total RNA from the cells and fresh mouse adipose tissues was isolated using TRIzol reagent (Takara, Tokyo, Japan) following the manufacturer’s instructions. The RT reaction was carried out using 1 μg of total RNA. Real-time PCR was performed with the Light Cycler 480 (Roche Applied Science, Indianapolis, IN) [14]. The following primer sequences were used: ATGL forward, 5′- GGATGAAAGAGCAGACGGGTAG −3′, and reverse, 5′-CGCAAGACAGTGGCACAGAG −3′, and β-actin forward, 5′-ACCCCAGCCATGTACGTAGC-3′, and reverse, 5′- GTGTGGGTGACCCCGTCTC-3′. β-actin was employed as an endogenous control for normalization.

### Immunofluorescence

Cells grown on coverslips were washed with PBS, fixed with 4% paraformaldehyde for 15 min, permeabilized with 0.2% Triton X-100 for 5 min and blocked using 10% goat serum in PBS for 30 min at room temperature. Cells were then incubated with primary antibodies (rabbit anti-ATGL, 1:100 dilution) in blocking buffer overnight at 4°C. Subsequently, the cells were incubated with secondary antibodies (FITC- or TRITC-conjugated, 1:50 dilution; Zhongshan Golden Bridge Biotechnology Co. Ltd) for 1 h at room temperature. The nuclei of the cells were visualized using mounting medium with DAPI. The fluorescence levels of the cells were determined using a confocal microscope (Axiovert 100M Zeiss, Zeppelinstrasse, Germany).

### Statistical analysis

Data are presented as the mean ± standard error of the mean (SEM). One-way analysis of variance (ANOVA) and T test were performed using the SPSS 13.0 software package. A value of P < 0.05 was considered to be statistically significant.

## Results

### ATGL is upregulated in epididymal adipose tissues of Tshr-/- mice

**Figure 1:**
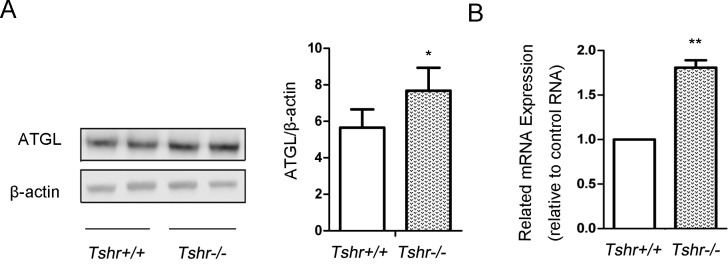
ATGL expression in visceral adipose tissues of Tshr-/- mice increased compared to that of Tshr+/+ mice. The epididymal adipose tissue was frozen in liquid nitrogen. Protein and mRNA were extracted according to the methods described before. (A) The protein expression levels of ATGL in the white adipose tissues of the *Tshr-/-* mice and *Tshr+/+* mice were detected by Western blotting. The relative ATGL protein levels were quantified by densitometry and normalized with β-actin. (B) The mRNA levels of ATGL in the white adipose tissue of the two types of mice were determined by real-time PCR and normalized with actin. The relative values representing the ATGL mRNA levels in the *Tshr-/-* mice are reported as fold changes relative to those of the *Tshr+/+* mice. The data are from 4 independent experiments and are presented as the mean ± SD. ** p < 0.01 *versus Tshr+/+* mice. * p < 0.05 *versus Tshr+/+* mice.

The function of TSH is mediated through the highly-specific receptor, TSHR [15]. To examine the effects of TSH on the TG lipolysis of adipocytes *in vivo*, we generated a *Tshr*-knockout mouse model. The ATGL expression in the visceral adipose tissues of the TSHR knockout mice (*Tshr-/-* mice) and WT mice (*Tshr+/+* mice) is differed. As shown in Fig. 1, the ATGL protein and mRNA levels significantly increased in the adipose tissue of the *Tshr-/-* mice compared to those of the *Tshr+/+* mice. These results indicated that ATGL expression in adipocytes was increased without the effect of TSH *in vivo*. To verify this finding *in vitro*, 3T3-L1 cells were cultured and differentiated to mature adipocytes and then treated with TSH.

### ATGL expression is increased during the differentiation process in 3T3-L1 preadipocytes

**Figure 2:**
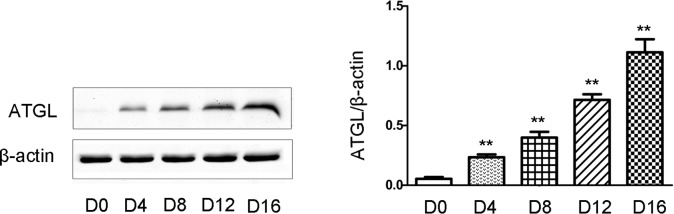
TSH stimulated ATGL protein expression in 3T3-L1 during the process of differentiation. Proteins were extracted from 3T3-L1 cells during the differentiation process every other day. Solubilized proteins were separated by SDS-PAGE and immunoblotted for ATGL and β-actin as indicated. Representative Western blot results are shown. The relative ATGL protein levels were quantified by densitometry and normalized with β-actin. The data are presented as the mean ± SD. ** p < 0.01 *versus* control group. Original magnification: 400×.

3T3-L1 preadipocytes were induced to differentiate as described in Materials and Methods. ATGL expression was determined by Western blotting. ATGL expression was observed to increase in the 3T3-L1 cells with increasing days, as shown in Fig. 2.

### TSH suppresses ATGL expression in differentiated 3T3-L1 adipocytes

**Figure 3:**
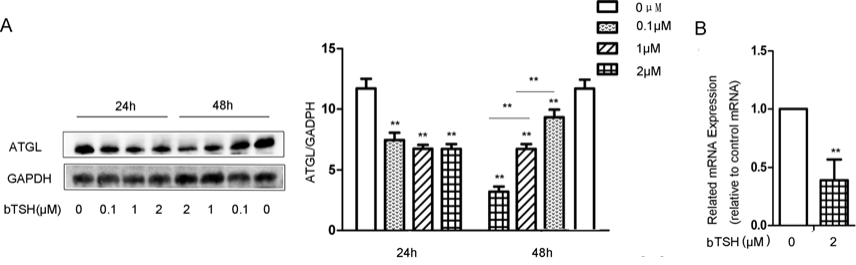
TSH decreased ATGL expression in mature differentiated cells. (A) On D12, the cells were treated with 0.1 μM bTSH, 1 μM bTSH or 2 μM bTSH for 24 h or 48 h in serum-starved DMEM. Proteins were separated by SDS-PAGE and immunoblotted for ATGL and GAPDH. Values are quantified by densitometry and normalized with GAPDH. Representative Western blot results are shown. (B) Total RNA was extracted from differentiated cells treated with 2 μM bTSH for 48 h in serum-free DMEM. ATGL mRNA levels were determined by real-time PCR and normalized with β-actin. Values are reported as the fold change relative to the control group. The data are from 3 independent experiments and are presented as the mean ± SD. ** p < 0.01 *versus* the control group. Original magnification: 400×.

Next, we explored the effect of TSH on ATGL expression in the differentiated 3T3-L1 cells. Fully differentiated 3T3-L1 cells were treated in the absence or presence of bTSH for 24 h and 48 h. ATGL expression was determined by Western blotting and RT-PCR, respectively. As shown in Fig. 3A, and Fig. 3B, TSH inhibited ATGL protein expression and mRNA expression. The reductions of ATGL expression in the 3T3-L1 cells were approximately 30%, 60% and 60% following treatment with 0.1 μM, 1 μM and 2 μM TSH, respectively. These results confirmed that TSH directly decreased the expression of ATGL in the mature 3T3-L1 cells.

### TSH-induced downregulation of ATGL in differentiated 3T3-L1 adipocytes is abolished by PKA inhibitor

**Figure 4:**
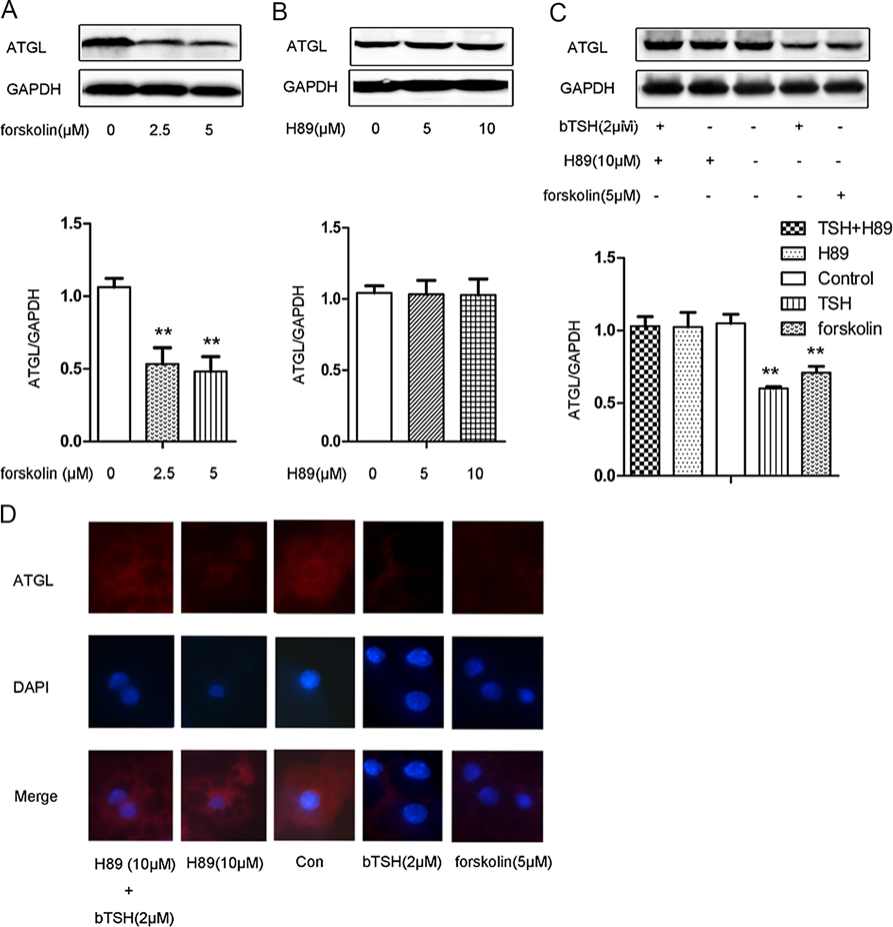
cAMP/PKA involved in TSH-induced downregulation of ATGL expression in differentiated 3T3-L1 cells. (A) On D12, cells were treated with 2.5 μM forskolin, 5 μM forskolin, or vehicle, respectively, for 24 h in serum-free DMEM. (B) On D12, the cells were stimulated with 5 μM H89, 10 μM H89 or vehicle, respectively, for 24 h in serum-free DMEM. (C) On D12, the cells were stimulated with 5 μM forskolin, 10 μM H89 or 2 μM bTSH. After 1 h, the cells from one of the two dishes pretreated with 10 μmol/L H89 were exposed to 2 μM bTSH. All cells were treated for 24 h in serum-free DMEM. Proteins were separated by SDS-PAGE and immunoblotted for ATGL and GAPDH, as indicated. Representative Western blot results are shown. (D) The visualization of ATGL by immunofluorescence staining (red). Nuclei were stained with DAPI (blue). Values are reported as the fold change relative to the control group. The data are presented as the mean ± SD. ** p < 0.01 *versus* the control group. Original magnification: 400 ×.

The cAMP/PKA pathway is the classic signaling pathway activated by TSH. To determine whether the effects of TSH on ATGL expression were mediated by this cAMP/PKA pathway, we treated the 3T3-L1 adipocytes with forskolin (adenylate cyclase activator) and H89 (PKA inhibitor). ATGL protein expression was determined by Western blotting. As shown in Fig. 4A, forskolin significantly decreased ATGL protein expression at all doses tested. H89 alone did not obviously affect ATGL expression (Fig. 4B). However, pretreatment with H89 abolished TSH-induced ATGL downregulation (Fig. 4C).

The above results were further confirmed by immunofluorescence staining (Fig. 4D).

## Discussion

In this study, we discovered a novel extra-thyroidal role of TSH in regulating TG metabolism. TSH inhibited ATGL expression, therefore inhibiting basal lipolysis in adipocytes. This activity involved the cAMP/PKA signaling pathway. Our findings indicate a potential role for TSH in the metabolism of TG in adipocytes and they may also aid in the elucidation of the relationship between subclinical hypothyroidism and obesity.

TSH is able to regulate TG metabolism in adipocytes, and a feedback loop between TSH and thyroid hormones exist within the human body [16]. Therefore, thyroid hormones activity must be ruled out to observe the direct effects of TSH on TG metabolism. We used *Tshr-/-* mice as our experimental model according to a previous study [13]. Thyroid hormone (T4) was administered to the animals after weaning to eliminate the influence of thyroid hormones. Serum total T4 levels were stably maintained in the *Tshr+/+* and *Tshr-/-* mice that were between 6 and 8 weeks old [17]. ATGL expression levels in the adipose tissues of the *Tshr-/-* mice increased compared with those of the wild-type mice. This suggests that ATGL expression in the mature adipocytes of *Tshr-/-* mice was not inhibited by TSH in the absence of TSHR. To confirm this finding *in vitro*, we cultured 3T3-L1 cells and treated the differentiated cells with TSH.

We demonstrated that ATGL expression increased during the process of differentiation, which is consistent with previous reports [3, 5–7]. In mature 3T3-L1 cells, ATGL expression was abolished following TSH treatment in a dose-dependent manner. The difference in ATGL expression between the two types of mice was not as distinctive as that observed in the differentiated 3T3-L1 cells. This may have occurred because TG metabolism in adipocytes is regulated by various factors in mice.

TG metabolism in adipocytes is regulated by several rate-limiting enzymes, including glycerol-3-phosphate acyltransferase 3 (GPAT3), which regulates the anabolism of TG and ATGL and hormone-sensitive lipase (HSL), which regulate catabolism of TG. Studies investigating the control of lipolysis have focused on HSL for decades. However, the non-obese phenotype of HSL knock-out mice [18] and the accumulation of diglycerides (DGs) in their adipose tissues [19] suggest that there may be one or more additional lipases present in adipose tissue that preferentially hydrolyze the first ester bond of the TG molecule. This discovery prompted increased research on ATGL activity. It is now accepted that ATGL is responsible for the initial step of lipolysis and is critical for the hydrolysis of TG during basal lipolysis [20]. The importance of ATGL in adipocyte lipid metabolism is also supported by reports of mice lacking ATGL that exhibit increased adipose masses and TAG depositions in their adipose tissues [9].

In an *in vitro* study, it was found that TSH has significant lipolytic effects in neonates, while in children and adults, its effects gradually decrease, and it is only present at unphysiological concentrations [21]. Gagnon et al have found that after treating mature 3T3-L1 cells with TSH at unphysiological concentrations for 4 h, medium FFA concentrations significantly increase, but this increase is not significant after 24 h. In addition, p-HSL expression was not found to change significantly at either 4 h or at 24 h. This study did not mention the long-term effects of TSH on mature adipocytes or the effects of TSH on ATGL [4]. Our study indicated that TSH had inhibitory effects on ATGL in mature 3T3-L1 cells, which confirmed the decreased ATGL expression inthe *Tshr-/-* mice. These findings represent novel evidence that contributes to the current understanding of the effects of TSH on basal lipolysis in adipocytes. Elgadi et al investigated the effects of TSH on white adipose tissue in mice with an adipose tissue-specific knockout of TSHR and found that basal lipolysis in TSHR-knockout adipocytes is higher than that in wild-type adipocytes on a per cell basis. However, this group did not explore the potential mechanism underlying their finding. Our study revealed that TSH decreased the expression of ATGL and therefore inhibited basal lipolysis in adipocytes, which may have partially accounted for the increased basal lipolysis observed in the TSHR-knockout adipocytes [22].

After combining with TSHR, TSH raises cAMP levels and stimulates the activity of PKA. This is one of the classic pathways by which TSH affects lipolysis. It is assumed that cAMP is the second messenger of the lipolytic response [23]. Studies have identified two phosphorylation sites, Ser-406 and Ser-430, in the C-terminal region of the ATGL molecule [24]. Ser-406 is a direct target of PKA, and its phosphorylation has been reported to be correlated with lipolytic activation in response to β-adrenergic stimulation [11, 12]. In the present study, we used forskolin to increase cAMP levels and H89 to selectively inhibit the cAMP-responsive kinase PKA. We found that forskolin decreased ATGL expression in the mature 3T3-L1 cells. In addition, the inhibitory effects of TSH on ATGL were abolished by exposure to H89. These results showed that the cAMP/PKA pathway was involved in the regulation of ATGL expression by TSH in the mature 3T3-L1 cells. However, the detailed underlying mechanism requires further exploration.

## Conclusions

The study revealed the novel role of TSH in decreasing the ATGL expression in the mature adipocytes of rodents. These findings suggest that TSH affects basal lipolysis. Further studies are needed to fully delineate the manner by which TSH regulates the metabolism of TG in human adipocytes. These studies may facilitate the development of therapeutic strategies for the treatment of obesity.

## References

[1] Martinez-Gonzalez C, Wang HL, Micklem BR, Bolam JP, Mena-Segovia J (2012) Subpopulations of cholinergic, GABAergic and glutamatergic neurons in the pedunculopontine nucleus contain calcium-binding proteins and are heterogeneously distributed. The European journal of neuroscience 35: 723–734. 10.1111/j.1460-9568.2012.08002.x 22356461

[2] Wanjia X, Chenggang W, Aihong W, Xiaomei Y, Jiajun Z, et al (2012) A high normal TSH level is associated with an atherogenic lipid profile in euthyroid non-smokers with newly diagnosed asymptomatic coronary heart disease. Lipids in health and disease 11: 44 10.1186/1476-511X-11-44 22448646 PMC3331821

[3] Lu S, Guan Q, Liu Y, Wang H, Xu W, et al (2012) Role of extrathyroidal TSHR expression in adipocyte differentiation and its association with obesity. Lipids in health and disease 11: 17 10.1186/1476-511X-11-17 22289392 PMC3285521

[4] Gagnon A, Antunes TT, Ly T, Pongsuwan P, Gavin C, et al (2010) Thyroid-stimulating hormone stimulates lipolysis in adipocytes in culture and raises serum free fatty acid levels in vivo. Metabolism: clinical and experimental 59: 547–553. 10.1016/j.metabol.2009.08.018 19846175

[5] Zimmermann R, Strauss JG, Haemmerle G, Schoiswohl G, Birner-Gruenberger R, et al (2004) Fat mobilization in adipose tissue is promoted by adipose triglyceride lipase. Science 306: 1383–1386. 10.1126/science.1100747 15550674

[6] Jenkins CM, Mancuso DJ, Yan W, Sims HF, Gibson B, et al (2004) Identification, cloning, expression, and purification of three novel human calcium-independent phospholipase A2 family members possessing triacylglycerol lipase and acylglycerol transacylase activities. The Journal of biological chemistry 279: 48968–48975. 10.1074/jbc.M407841200 15364929

[7] Villena JA, Roy S, Sarkadi-Nagy E, Kim KH, Sul HS (2004) Desnutrin, an adipocyte gene encoding a novel patatin domain-containing protein, is induced by fasting and glucocorticoids: ectopic expression of desnutrin increases triglyceride hydrolysis. The Journal of biological chemistry 279: 47066–47075. 10.1074/jbc.M403855200 15337759

[8] Zechner R, Kienesberger PC, Haemmerle G, Zimmermann R, Lass A (2009) Adipose triglyceride lipase and the lipolytic catabolism of cellular fat stores. Journal of lipid research 50: 3–21. 10.1194/jlr.R800031-JLR200 18952573

[9] Haemmerle G, Lass A, Zimmermann R, Gorkiewicz G, Meyer C, et al (2006) Defective lipolysis and altered energy metabolism in mice lacking adipose triglyceride lipase. Science 312: 734–737. 10.1126/science.1123965 16675698

[10] Huijsman E, van de Par C, Economou C, van der Poel C, Lynch GS, et al (2009) Adipose triacylglycerol lipase deletion alters whole body energy metabolism and impairs exercise performance in mice. American journal of physiology Endocrinology and metabolism 297: E505–513. 10.1152/ajpendo.00190.2009 19491295

[11] Mason RR, Meex RC, Lee-Young R, Canny BJ, Watt MJ (2012) Phosphorylation of adipose triglyceride lipase Ser(404) is not related to 5′-AMPK activation during moderate-intensity exercise in humans. American journal of physiology Endocrinology and metabolism 303: E534–541. 10.1152/ajpendo.00082.2012 22713505

[12] Pagnon J, Matzaris M, Stark R, Meex RC, Macaulay SL, et al (2012) Identification and functional characterization of protein kinase A phosphorylation sites in the major lipolytic protein, adipose triglyceride lipase. Endocrinology 153: 4278–4289. 10.1210/en.2012-1127 22733971

[13] Marians RC, Ng L, Blair HC, Unger P, Graves PN, et al (2002) Defining thyrotropin-dependent and-independent steps of thyroid hormone synthesis by using thyrotropin receptor-null mice. Proceedings of the National Academy of Sciences of the United States of America 99: 15776–15781. 10.1073/pnas.242322099 12432094 PMC137792

[14] Schmittgen TD, Livak KJ (2008) Analyzing real-time PCR data by the comparative C(T) method. Nature protocols 3: 1101–1108. 10.1038/nprot.2008.73 18546601

[15] Dumont JE (1971) The action of thyrotropin on thyroid metabolism. Vitamins and hormones 29: 287–412. 10.1016/S0083-6729(08)60051-5 4400011

[16] Garg A, Vanderpump MP (2013) Subclinical thyroid disease. British medical bulletin 107: 101–116. 10.1093/bmb/ldt024 23919951

[17] Wang T, Xu J, Bo T, Zhou X, Jiang X, et al (2013) Decreased fasting blood glucose is associated with impaired hepatic glucose production in thyroid-stimulating hormone receptor knockout mice. Endocrine journal 60: 941–950. 10.1507/endocrj.EJ12-0462 23665701

[18] Zimmermann R, Haemmerle G, Wagner EM, Strauss JG, Kratky D, et al (2003) Decreased fatty acid esterification compensates for the reduced lipolytic activity in hormone-sensitive lipase-deficient white adipose tissue. Journal of lipid research 44: 2089–2099. 10.1194/jlr.M300190-JLR200 12923228

[19] Haemmerle G, Zimmermann R, Hayn M, Theussl C, Waeg G, et al (2002) Hormone-sensitive lipase deficiency in mice causes diglyceride accumulation in adipose tissue, muscle, and testis. The Journal of biological chemistry 277: 4806–4815. 10.1074/jbc.M110355200 11717312

[20] Langin D, Dicker A, Tavernier G, Hoffstedt J, Mairal A, et al (2005) Adipocyte lipases and defect of lipolysis in human obesity. Diabetes 54: 3190–3197. 10.2337/diabetes.54.11.3190 16249444

[21] Marcus C, Ehren H, Bolme P, Arner P (1988) Regulation of lipolysis during the neonatal period. Importance of thyrotropin. The Journal of clinical investigation 82: 1793–1797. 10.1172/JCI113793 3183066 PMC442750

[22] Elgadi A, Zemack H, Marcus C, Norgren S (2010) Tissue-specific knockout of TSHr in white adipose tissue increases adipocyte size and decreases TSH-induced lipolysis. Biochemical and biophysical research communications 393: 526–530. 10.1016/j.bbrc.2010.02.042 20152797

[23] Janson A, Karlsson FA, Micha-Johansson G, Bolme P, Bronnegard M, et al (1995) Effects of stimulatory and inhibitory thyrotropin receptor antibodies on lipolysis in infant adipocytes. The Journal of clinical endocrinology and metabolism 80: 1712–1716. 10.1210/jc.80.5.1712 7745024

[24] Bartz R, Zehmer JK, Zhu M, Chen Y, Serrero G, et al (2007) Dynamic activity of lipid droplets: protein phosphorylation and GTP-mediated protein translocation. Journal of proteome research 6: 3256–3265. 10.1021/pr070158j 17608402

